# Aminergic Control of Social Status in Crayfish Agonistic Encounters

**DOI:** 10.1371/journal.pone.0074489

**Published:** 2013-09-18

**Authors:** Yuto Momohara, Akihiro Kanai, Toshiki Nagayama

**Affiliations:** 1 Division of Biology, Graduate School of Science and Engineering, Yamagata University, Yamagata, Japan; 2 Department of Biology, Faculty of Science, Yamagata University, Yamagata, Japan; University of Iowa, United States of America

## Abstract

Using pairings of male crayfish *Procambarus clarkii* with a 3–7% difference in size, we confirmed that physically larger crayfish were more likely to win encounters (winning probability of over 80%). Despite a physical disadvantage, small winners of the first pairings were more likely to win their subsequent conflicts with larger naive animals (winning probability was about 70%). By contrast, the losers of the first pairings rarely won their subsequent conflicts with smaller naive animals (winning probability of 6%). These winner and loser effects were mimicked by injection of serotonin and octopamine. Serotonin-injected naive small crayfish were more likely to win in pairings with untreated larger naive crayfish (winning probability of over 60%), while octopamine-injected naive large animals were beaten by untreated smaller naive animals (winning probability of 20%). Furthermore, the winner effects of dominant crayfish were cancelled by the injection of mianserin, an antagonist of serotonin receptors and were reinforced by the injection of fluoxetin, serotonin reuptake inhibitor, just after the establishment of social order of the first pairings. Injection of octopamine channel blockers, phentolamine and epinastine, by contrast, cancelled the loser effects. These results strongly suggested that serotonin and octopamine were responsible for winner and loser effects, respectively.

## Introduction

The establishment of status-dependent dominance hierarchy through conflict between conspecifics is essential for territorial animals to maintain their social stability. Together with physical asymmetries of body size, sexes and prior residence, social experiences influence dominance hierarchy formation and shape emerging social structures. A previously winning experience increases the winning probability of the next agonistic encounter, whereas a previous losing experience has the opposite effect. These winner and loser effects have been widely described in both vertebrates [1], [2], [3], [4], [5], [6] and arthropods [7], [8]. [9], [10], [11], [12], [13], [14]. Surprisingly however, few studies have focused on clarifying the neural mechanisms underlying the winner and loser effects.

To assume that winning increases the level of aggression while losing decreases aggressive motivation would be reasonable and evidence shows that the winning experience in crickets enhances aggressiveness [15]. In vertebrates, androgen, noradrenaline, and dopamine are known to promote aggressive behavior [4], [16], [17]. In crickets, the phenol analogue of noradrenaline, octopamine increases aggressive motivation [18]. By contrast, serotonin enhances aggression in crustaceans [19], [20], although it suppresses aggressiveness in vertebrates [21]. Injection of serotonin elicits a dominant-like posture in both lobsters and crayfish [22], [23] and an aggressive display in squat lobsters [24]. Furthermore, octopamine in crustaceans causes submissive posture [22]. Despite the effects known of amines on aggressive motivation, the mechanisms underlying winner and loser effects are still poorly understood. In crayfish and crabs, the injection of serotonin or octopamine into dominant and subordinate animals modifies aggressiveness during the following encounters but fails to reverse previous hierarchical rank in [25], [26], [27], [28].

The repertories and processes during agonistic encounters of crayfish have been analyzed in detail [29], [30], [31] and the relevant changes in both agonistic and non-agonistic behaviors of individuals according to the acquired social order have also been well characterized [32], [33], [34]. These findings as well as the above-mentioned aminergic modulation [35] suggest crayfish represent an ideal animal in which to study agonistic encounters behaviorally, physiologically and pharmacologically. In this study using pairings of crayfish with a 3–7% difference in body size, we have characterized the role of serotonin and octopamine quantitatively. Small crayfish injected with serotonin tended to win encounters while larger crayfish injected with octopamine were frequently beaten. The winner and loser effects disappeared when serotonergic and octopaminergic antagonists were injected.

## Materials and Methods

### Animals

Adult male crayfish, *Procambarus clarkii* Girard (6–9 cm body length from rostrum to telson and 10–23 g body weight) were used in all experiments. Crayfish were purchased from a commercial supplier in Okayama, Japan and maintained individually in separate opaque containers of 19 (width)×33 (length)×15 (height) cm filled with water to a depth of 10 cm for at least 30 days. Each crayfish was fed equal amounts of small food pellets once a week and was last fed at least 5 days before pairings. Crayfish were maintained under a 12 hr: 12 hr light-dark cycle. Experimental trials were carried out in a dimly lit laboratory at a room temperature of approximately 23°C. Crayfish that molted within a week before experiments were not used in this study.

### First Pairings

The day before pairing, the body length (from rostrum to telson) and wet mass of each crayfish were measured. Two crayfish with a length difference between 3–7% and with a mass difference between 3–12% were selected and paired in a new opaque container of 26 (width)×38 (length)×24 (height) cm filled with water to about half depth. Larger crayfish thus had a longer body length and heavier body mass than smaller opponents. Prior to each trial, an opaque plastic barrier was placed in the center of the tank separating it into two areas. A single crayfish was placed on each side of this barrier and allowed to acclimate for at least 10 min before the divider was removed.

The agonistic bouts of the crayfish were recorded using a video camera (Victor GZ-MG330-S, Japan) mounted on a tripod above the container for 45 min. The behavior of each crayfish was analyzed using single frame measurement to construct an ethogram of each second of the encounter. Behavioral acts that occurred during agonistic bouts were categorized as one of seven types: attack, fight, contact, approach, retreat, tailflip and neutral (modified from [31]). Before determination of dominance status, the crayfish that initiated the approach was frequently beaten in the following bouts by their opponents. The winner and loser relationship was determined with several fights. After the establishment of a dominance order, subordinate crayfish showed a retreat or tailflip following the dominant’s attack that was a rapid approach with both chelae raised. No fight was observed at this stage. We determined the dominance order of paired crayfish when the subordinate crayfish showed a retreat or tailflip following the dominant’s attack on at least three times in succession [31]. After 45 min of pairing, dominant and subordinate crayfish were re-isolated for a second session of pairing on the next day.

### Second Pairings

Since dominance order is maintained more than a week [36], dominant or subordinate crayfish on the first day were paired with different opponents of one of naive, dominant or subordinate crayfish with larger or smaller body length and body mass on the next day. In all pairings, two crayfish with a length difference between 3–8% and with a mass difference between 3–11% were selected. We defined crayfish as NL (naive large) that is a newcomer without previous pairing and larger in size than the opponent, NS (naive small) that is a newcomer without previous pairing and smaller in size than the opponent, DL (dominant large) that was a winner of a pairing on the previous day and larger in size than the present opponent, DS (dominant small) that was a winner of a previous pairing and smaller in size than the present opponent, SL (subordinate large) that was a loser of a pairing on the previous day and larger in size than the present opponent, and SS (subordinate small) that was a loser of a previous pairing and smaller in size than the present opponent. Using different combination of these crayfish, we have examined the effect of physical difference and previous social experience during agonistic encounters.

### Amine Injection

The following pharmacological agents were obtained from Sigma (St Louis, MO, USA): biogenic amines, serotonin creatinine sulfate complex (5 HT), (±)-octopamine hydrochloride (OA) and their precursors, 5-hydroxy-L-tryptophan (5 HTP), tyramine hydrochloride (TA), as well as their receptor antagonists, mianserin hydrochloride (mian) [37], phentolamine hydrochloride (phen) [38], epinastine hydrochloride [39], and serotonin reuptake inhibitor, fluoxetin hydrochloride [26]. These drugs were dissolved in physiological saline [40] and made up to the required concentration prior to each experiment with the exception of 5 HTP and mianserin that was dissolved in distilled water to 1 mM first and then dissolved in saline to make up to the required concentration, and fluoxetin that was dissolved in DMSO to 10 mM first and then dissolved in saline to make up to 10 µM in concentration. Injection of drugs ( = 1 ml in volume) was made into the pericardial sinus from the dorsal carapace within the caudal third of the pericard to avoid damaging the underlying heart through a 27-3/4 gauge needle. This injection point was determined according to [41]. The amount of injected drugs was 10 nmol if a crayfish was injected with 1 ml of 10 µM drug solution.

### Statistic Analyses

The winning probability was determined by the number of animals that won the pairings/total number of agonistic bouts. The differences in winning probability were analyzed statistically using a Fisher’s exact test and Binomial Test, while the decision time for dominance order to be established was analyzed using a log rank test and the number and duration of fights was analyzed using a Student’s t test if data were normally distributed, or a Mann-Whitney Rank Sum Test if not. Statistical analyses were carried out using SigmaPlot v11 and R 2.14.1.

## Results

### Physical Effects during Agonistic Bouts

**Figure 1:**
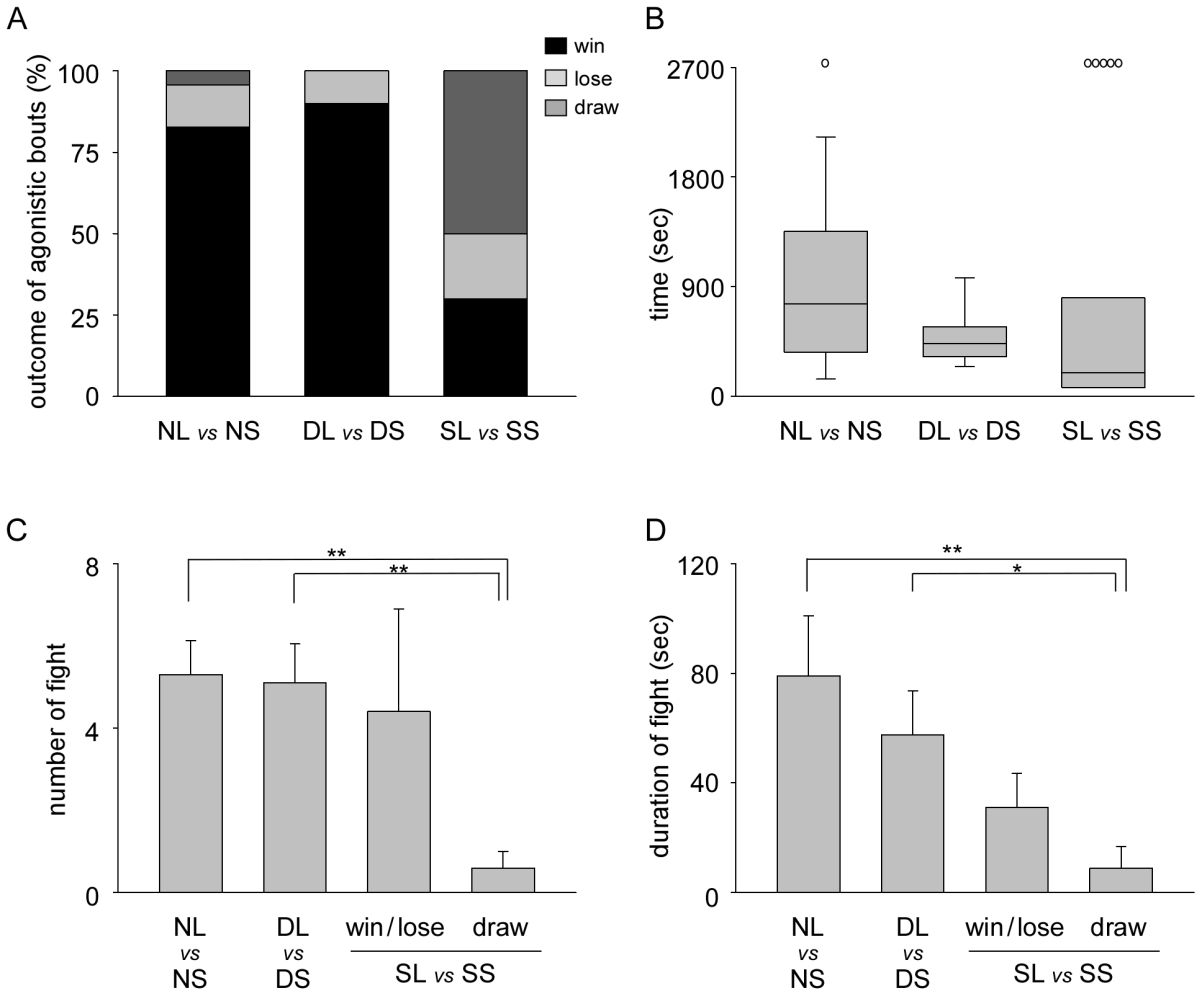
Effect of physical size difference on crayfish agonistic bouts. A, Winning rate of larger crayfish during agonistic bouts in pairings with smaller crayfish. The percentages of outcomes of agonistic bouts of larger crayfish are plotted as wins (black), losses (light grey) and draws (dark grey). Left: pairings between naive large (NL) and naive small (NS) animals, Middle: pairings between dominant large (DL) and dominant small (DS) animals, and Right: pairings between subordinate large (SL) and subordinate small (SS) animals. B, The time taken for the dominant-subordinate relationship to form. Box plots show median (solid black line), interquartile range (box length), and minimum and maximum values (error bars). Open circles indicate pairs that did not show clear dominant-subordinate relationship after 45 mins of pairings. C, The number of fights between pairings during the first 30 min agonistic bouts. D, The average duration of individual fights of pairings during the first 30 min of agonistic bouts. In C and D, the number of fights and the average duration of individual fights in subordinate pairings are separately plotted whether the dominant-subordinate relationship was determined or not. Asterisks in C and D indicate that the number and duration of responses differed significantly (Mann-Whitney rank sum test, *p<0.05, **p<0.01).

After pairing of two naive crayfish, that were isolated individually for more than one month in separate tanks, in the fighting arena, they started agonistic behavior, e.g. approach and fighting. In 23 pairings, crayfish with a greater body length and heavier body mass won in 19 pairings. The smaller crayfish won in three pairings, while in the remaining pairing no winner was found within a 45 min period (Fig. 1A left). The winning percentage of large crayfish was 83% with larger crayfish likely to win (binomial test, *p = *0.002). After 45 min of pairing, dominant and subordinate crayfish were again re-isolated. On the following day, crayfish that became dominant after the first agonistic bout were paired with another dominant crayfish, while crayfish that became subordinate after the first agonistic bout were paired with another subordinate crayfish. In 10 dominant pairings, the larger crayfish (3–8% longer and 3–11% heavier than small opponents) won in 9 pairings (Fig. 1A middle) that were more likely to win (binomial test, *p* = 0.021). By contrast, in 10 subordinate pairings, the larger crayfish (3–5% longer and 4–10% heavier than small opponents) won in 3 pairings and the smaller crayfish won in 2 pairings. The crayfish that initiated the approach first became dominant in 4 out of 5 pairings. In the remaining 5 pairings, a dominant-subordinate relationship was not clearly established within a 45 min test period (Fig. 1A right). The winning probability of larger crayfish in subordinate pairings was significantly lower than that of larger crayfish, in both naive and dominant pairings (Fisher’s exact test, *p* = 0.006 with naive pairings and *p* = 0.020 with dominant pairings).

Figure 1B shows the length of time it took for the dominant-subordinate relationship to form. The median of each group was 760 sec in naive pairings (n = 22 out of 23 pairings), 432 sec in dominant pairings (n = 10) and 195 sec in subordinate pairings (n = 5 out of 10 pairings). Crayfish in dominant pairings formed a hierarchy more rapidly than crayfish in naive pairings (log-rank test, *p* = 0.009) and crayfish in subordinate pairings if the results of draw pairs are included (log-rank test, *p = *0.034). The total number of fights during agonistic encounters within the first 30 min was 5.3±0.8 in naive pairings and 5.1±0.9 in dominant pairings (Fig. 1C), with the average duration of individual fights being 79.1±21.9 s in naive pairings and 57.6±16.0 s in dominant pairings (Fig. 1D) and not statistically different (Mann-Whitney rank sum test, *p* = 0.766 and 0.652, respectively). Thus, in dominant pairings, crayfish fought repeatedly but for shorter duration. The total number of fights between subordinate pairings in which a dominant-subordinate relationship was established was 4.4±2.5 while the average duration of individual fights was 31.1±12.3 s (Fig. 1C,D) and was also not statistically different to naive and dominant pairings. In pairings between subordinate animals without establishment of dominant-subordinate relationship, crayfish avoided physical encounterings. The total number of fights was 0.6±0.4 (Fig. 1C), and was significantly fewer than naive and dominant pairings (Mann-Whitney rank sum test, *p* = 0.002 with naive pairings and *p* = 0.007 with dominant pairings), but not different to subordinate pairings that had established dominant-subordinate relationship (Mann-Whitney rank sum test, *p = *0.151). The average duration of individual fights was 8.8±8.1 s (Fig. 1D) that was significantly shorter than naive and dominant pairings (Mann-Whitney rank sum test, *p* = 0.004 with naive pairings and *p* = 0.023 with dominant pairings) but was not different from subordinate pairings that had established dominant-subordinate relationship (Mann-Whitney rank sum test, *p = *0.169). Thus, a physical advantage affected the outcome of agonistic bouts in naive and dominant pairings but did not influence significantly the outcome of agonistic bouts in subordinate pairings.

### Winner and Loser Effects during Agonistic Bouts

**Figure 2:**
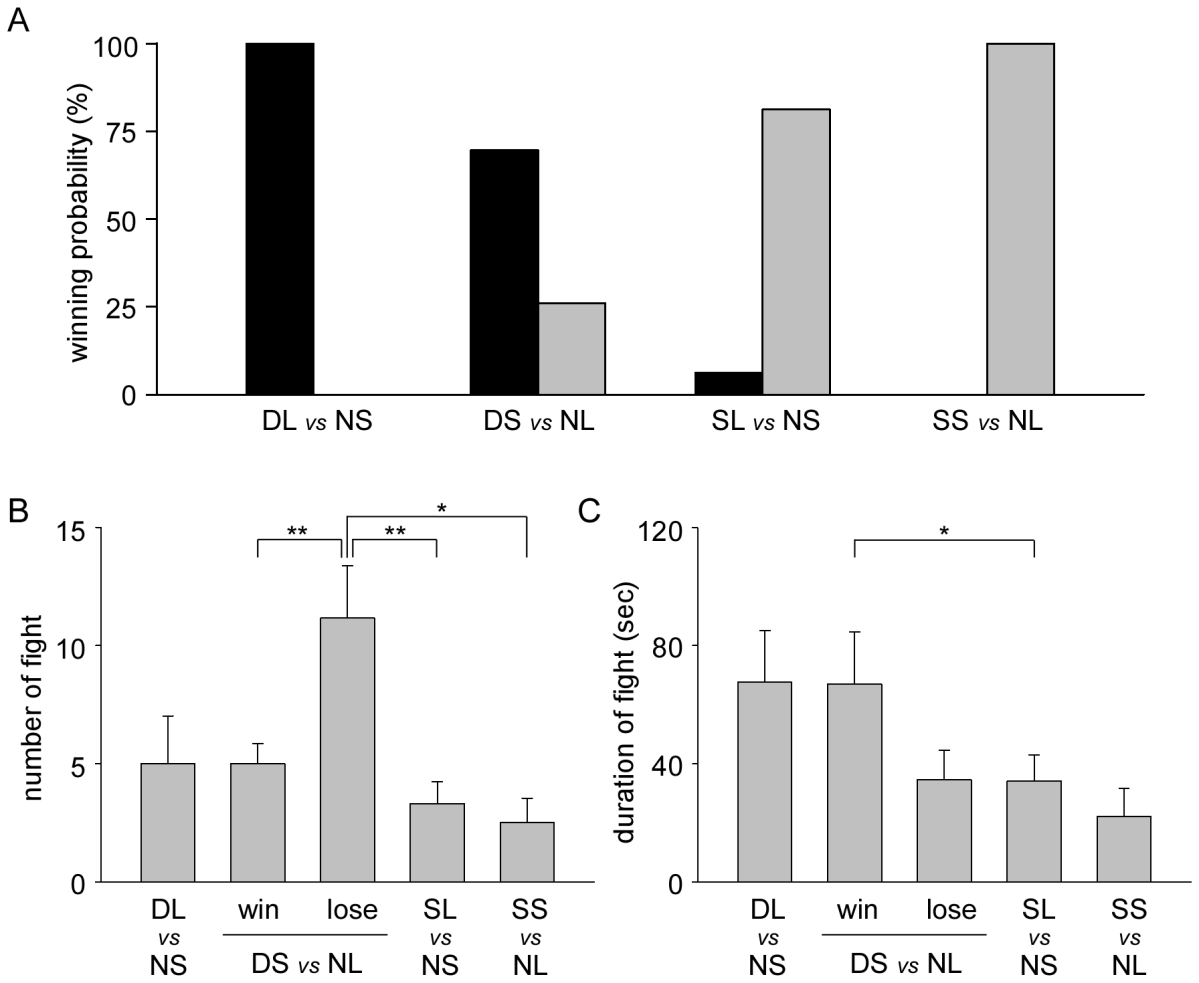
Effect of previous social experiences on agonistic bouts after 24 hrs. A, Winning rate of crayfish during agonistic bouts. Dominant and subordinate crayfish were paired with un-experienced naive crayfish with a different body size on the next day. The percentages of winning outcomes of agonistic bouts of each group are plotted. From left to right: pairings between dominant large and naive small animals, dominant small and naive large animals, subordinate large and naive small animals, and subordinate small and naive large animals. No naive small crayfish paired with dominant large animals (Left) or subordinate small crayfish paired with naive large animals (Right) win. B, The number of fights of pairings during the first 30 min of agonistic bouts. C, The average duration of individual fights of pairings during the first 30 min of agonistic bouts. In B and C, the number of fights and the average duration of individual fights between dominant small and naive large crayfish are plotted separately whether dominant small animals win or lose. Asterisks in B and C indicate that the number and duration of responses differed significantly (Mann-Whitney rank sum test, *p<0.05, **p<0.01).

Since the results shown in Figure 1 suggested that the aggressiveness of dominant and subordinate crayfish is different, we have analyzed the effect of hysteresis of previous agonistic bouts. The day after the first pairings, crayfish that became dominant or subordinates were paired with unfamiliar naive crayfish (Fig. 2A). When dominants were paired with smaller naive crayfish in 5 pairing, the dominant large crayfish won in all encounters. In 23 pairings between dominant small and naive large crayfish, the dominant small crayfish won in 70% (16 of 23) of pairings while the naive large crayfish won in only 26% (6 of 23) of pairings. A binomial test showed there was no difference between the likelihood of dominant small crayfish winning compared to naive large crayfish (*p* = 0.093), but they were more likely to win against naive small crayfish (cf. Fig. 1A and Table 1A, Fisher’s exact test, *p*<0.001). Naive large crayfish beat smaller subordinate crayfish in all 5 pairings. In 16 pairings between subordinate large and naive small animals, the larger subordinate crayfish won in only one pairing ( = 6%) while the smaller naive opponents won in 81% (13 of 16) of pairings. Naive small crayfish were statistically more likely to win against subordinate large animals (binomial test, p = 0.021), and crayfish that were larger in size but became subordinates in a previous pairing were beaten more frequently than naive large crayfish (cf. Fig. 1A and Table 2A, Fisher’s exact test, *p = *0.001). Thus, social experience on the previous day affected significantly the outcome of the following agonistic bouts.

**Table 1:** Summary of the outcome of agonistic encounters in smaller crayfish.

	animal	opponent	outcome of pairings				Fisher’s exact test	
			win	lose	draw	winningrate(%)	againstNS	againstDS
A	Naive Small (NS)	Naive Large	3	19	1	13.04	–	0.0002216
	Dominant Small (DS)	Naive Large	16	6	1	69.57	0.0002216	–
	Subordinate Small (SS)	Naive Large	0	5	0	0	1	0.0080586
B	saline injected into Naive Small 10 m before pairing	Naive Large	3	11	1	20	0.663208	0.0069406
	0.1 µM 5 HT injected into NS 10 m before pairing	Naive Large	3	9	0	25	0.391176	0.0298215
	0.5 µM 5 HT injected into NS 10 m before pairing	Naive Large	5	8	1	35.71	0.215234	0.0856527
	1.0 µM 5 HT injected into NS 10 m before pairing	Naive Large	10	6	0	62.5	0.0020281	0.736324
	2.0 µM 5 HT injected into NS 10 m before pairing	Naive Large	7	4	0	63.64	0.0047850	1
	10 µM 5 HTP injected into NS 10 m before pairing	Naive Large	5	0	2	71.43	0.0066607	1
	25 µM phentolamine injected into NS 10 m before pairing	Naive Large	3	7	0	30	0.336415	no test
C	saline injected into DS after social rank formation	Naive Large	9	3	0	75	0.0004872	1
	10 µM mianserin injected into DS after social rank formation	Naive Large	4	6	0	40	0.160526	0.139342
	20 µM mianserin injected into DS after social rank formation	Naive Large	4	9	0	30.77	0.224999	0.0379399
	50 µM mianserin injected into DS after social rank formation	Naive Large	2	8	0	20	0.6269	0.0199944
	25 µM phentolamine injected into DS after social rank formation	Naive Large	4	1	0	80	0.0076923	1

**Table 2:** Summary of the outcome of agonistic encounters in larger crayfish.

	animal	opponent	outcome of pairings				Fisher’s exact test	
			win	lose	draw	winning rate(%)	against NL	against SL
A	Naive Large (NL)	Naive Small	19	3	1	82.61	–	0.0012820
	Dominant Large (DL)	Naive Small	5	0	0	100	1	0.0002948
	Subordinate Large (SL)	Naive Small	1	13	2	6.25	0.0012820	–
B	saline injected into Naive Large 10 min before pairing	Naive Small	9	1	2	75	0.669741	0.0002732
	0.1 µM OA injected into NL 10 min before pairing	Naive Small	5	2	1	62.5	0.334589	0.0068649
	0.5 µM OA injected into NL 10 min before pairing	Naive Small	6	3	1	60	0.205421	0.0052903
	1.0 µM OA injected into NL 10 min before pairing	Naive Small	2	8	0	20	0.0011736	0.538462
	0.1 µM TA injected into NL 10 min before pairing	Naive Small	3	6	1	30	0.0059154	0.264214
C	20 µM mianserin injected into NL 10 min before pairing	Naive Small	4	3	3	40	0.0348716	no test
	20 µM mianserin injected into NL 1 HR before pairing	Naive Small	5	5	0	50	0.0895054	no test
D	saline injected into SL after social rank formation	Naive Small	1	11	0	8.33	0.0000333	1
	10 µM phentolamine injected into SL after social rank formation	Naive Small	2	7	1	20	0.0011736	0.538462
	25 µM phentolamine injected into SL after social rank formation	Naive Small	5	5	0	50	0.0895054	0.0184251
	20 µM mianserin injected into SL after social rank formation	Naive Small	2	8	0	20	0.0011736	0.538462

In pairings between dominant small and naive large animals, the average length of time it took for dominant-subordinate relationship to be determined was 745.2±119.6 s when dominant small animals won, and was 899.5±148.3 s when dominant small crayfish were beaten by naive large crayfish. The average length of decision time of pairings between subordinate large losers and naive small winners was 407.4±61.1 s that was significantly shorter than for dominant small losers and naive small losers (Fig. 1A) in pairings with larger naive opponents (log-rank test, *p* = 0.003). The total number of fights during agonistic encounters within the first 30 min and the average duration of individual fights of each pairings are shown in Figure 2B and 2C, respectively. The number of fights in the pairings between dominant large and naive small animals was 5.0±2.0 and the average duration of an individual fight was 67.7±17.4 s, and were similar to those of pairings between naive large and naive small crayfish shown in Figure 1. The number of fights in the pairings between subordinate large and naive small animals was 3.3±0.9 and the average duration of an individual fight was 34.1±8.8 s, while the number of fights in the pairings between subordinate small and naive large animals was 2.5±1.0 and the average duration of an individual fight was 22.1±9.6 s. There were no significant difference between pairings, but statistically different from the pairings between naive large and naive small animals (Mann-Whitney rank sum test, *p* = 0.045 with number of fights and *p* = 0.026 with the average duration of an individual fight). In the pairings between dominant small and naive large crayfish, the mean number of fights was 5.0±0.8 and the average duration of an individual fight was 66.9±17.9 s when dominant small crayfish beat naive large animals. Those were similar with the pairings between dominant large and naive small animals. However, when dominant small crayfish were beaten by naive large animals, the number of fights increased to 11.2±2.2 and was significantly higher than other pairings except for pairings between dominant large and naive small animals (t-test, *p* = 0.004 against winning dominant small *vs* losing naive large pairs, Mann-Whitney rank sum test, *p* = 0.004 against subordinate large *vs* naive small pairs, t-test, *p* = 0.017 against subordinate small *vs* naive large pairs, and Mann-Whitney rank sum test, *p* = 0.021 against naive large *vs* naive small pairs). The average duration of an individual fight of losing dominant small animals was short to 34.7±10.0 s, but there was no significant difference with other pairings.

### Effect of Serotonin Injection

**Figure 3:**
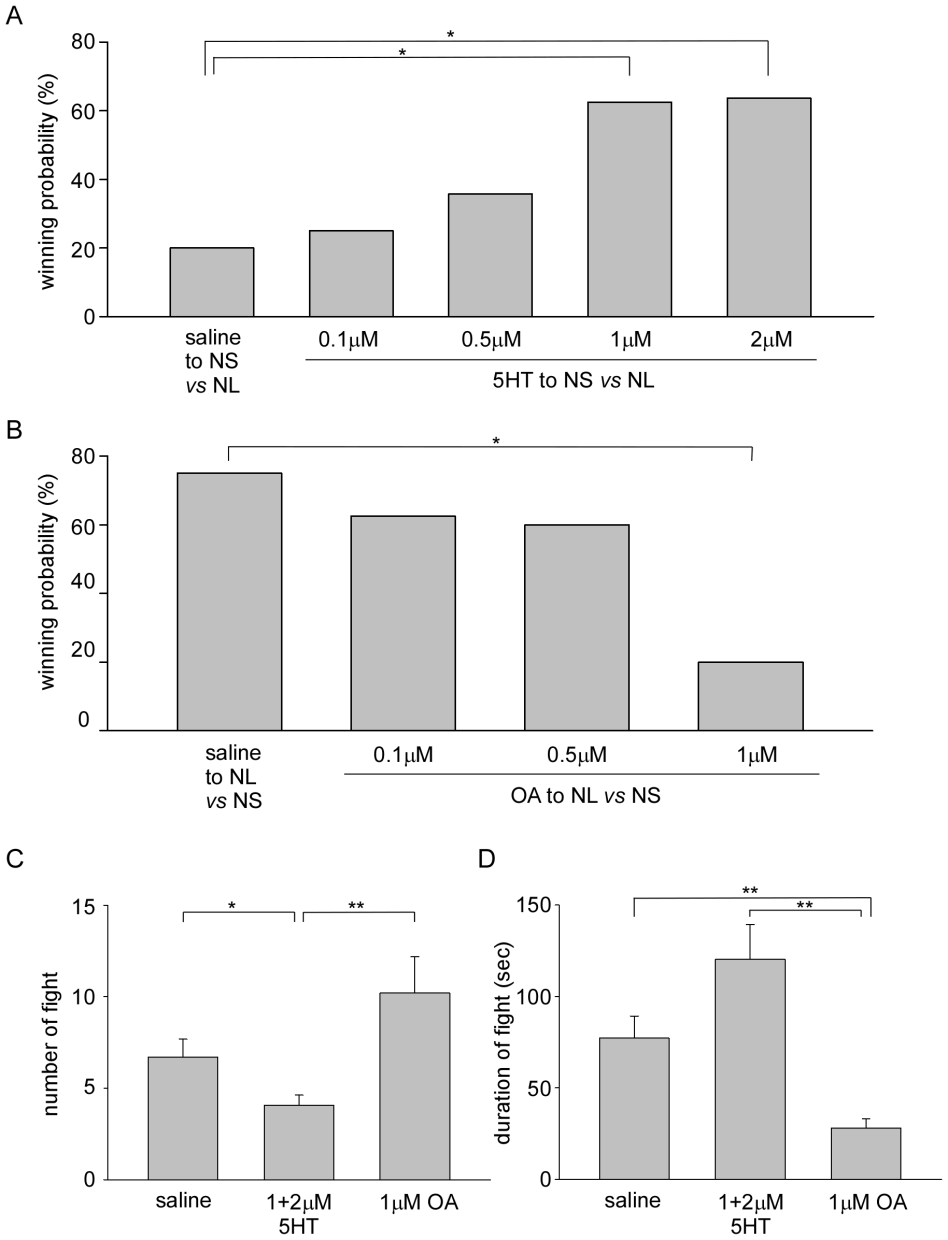
Effects of serotonin and octopamine upon agonistic bouts. A, Winning rate of naive small crayfish injected serotonin at various concentrations 10-injected smaller crayfish are plotted. B, Winning rate of naive large crayfish injected octopamine at various concentrations 10 min prior to the pairings with naive small animals. The percentages of winning outcomes of agonistic bouts of octopamine-injected larger crayfish are plotted. Asterisks in *A* and *B* indicate that the winning rate of small crayfish (A) or large crayfish (B) differed significantly (Fisher’s exact test, *p<0.05). C, The number of fights of pairings during the first 30 min of agonistic bouts. D, The average duration of individual fights of pairings during the first 30 min of agonistic bouts. In C and D, data from saline-injected small (A) and large crayfish (B) are summed together as a saline group, and 1 and 2 µM serotonin-injected small crayfish are also summed. Asterisks in C and D indicate that the number and duration of responses differed significantly (Mann-Whitney rank sum test, *p<0.05, **p<0.01).

Serotonin at various concentrations was injected into the pericardial sinus of naive small crayfish 10 min prior to pairings with naive large animals (Fig. 3A). When saline alone was injected, treated crayfish won in 20% (3 of 15) of pairings while larger untreated crayfish won in 73% (11 of 15) of pairings. The remaining one pair showed no dominant-subordinate relationship after 45 min of pairing. Thus, naive large crayfish were likely to win (binomial test, *p* = 0.035). The winning probability of saline-injected small crayfish in pairings with naive large crayfish was not statistically different from that of naive small crayfish in pairings with naive large crayfish (Table 1B, Fisher’s exact test, *p* = 0.663), while it was significantly lower than that of dominant small crayfish in pairings with naive large crayfish (Table 1B, Fisher’s exact test, *p* = 0.007).

The winning probability of treated small crayfish gradually increased depending on the concentration of injected serotonin. For example, 25% (3 of 12) of small crayfish won when 0.1 µM serotonin was injected, and 36% (5 of 14) of small crayfish won when 0.5 µM serotonin was injected. The winning probabilities of these small crayfish were not statistically different from those of saline-injected small crayfish or untreated small crayfish (Table 1B, Fisher’s exact test, *p* = 1.0 in 0.1 µM serotonin-injected small crayfish and *p* = 0.427 in 0.5 µM serotonin-injected small crayfish against saline-injected small crayfish and *p* = 0.391 and 0.215 against untreated small crayfish) but different from those of dominant small crayfish shown in Figure 2A (Table 1B, Fisher’s exact test, *p* = 0.030 in 0.1 µM serotonin-injected animals and *p* = 0.086 in 0.5 µM serotonin-injected animals). When 1 µM serotonin was injected into smaller crayfish, they won in 63% (10 of 16) of pairings. Furthermore, serotonin-treated smaller crayfish won in 64% (7 of 11) of pairings when 2 µM serotonin was injected. The winning probabilities of 1 µM and 2 µM serotonin-injected small crayfish increased significantly compared to saline-injected small crayfish (Fisher’s exact test, *p* = 0.029 in 1 µM serotonin-injected animals and *p* = 0.043 in 2 µM serotonin-injected animals in pairings with dominant small crayfish). Thus, serotonin-injected small crayfish won in over 60% in pairings against physically advantaged larger crayfish as dominant small animals shown in Figure 2A (Table 1B, Fisher’s exact test, *p* = 0.736 in 1 µM serotonin-injected animals and *p* = 1.0 in 2 µM serotonin-injected animals in pairings with dominant small crayfish, while *p* = 0.002 and 0.005 against no treated naive small crayfish, respectively). Since crayfish frequently showed unnatural postures or irregular movements when serotonin at 5 µM or higher concentrations were injected, we did not examine the effect of serotonin of more than 5 µM in this paper.

In 7 animals, 10 µM 5 HTP, a precursor of serotonin, was injected into naive small crayfish 10 min prior to the pairings with larger naive crayfish. Five 5HTP-injected animals won and the remaining two pairs showed no dominant-subordinate relationship after 45 min of pairing. The winning probability of 5HTP-injected small crayfish was 71%, and was significantly higher than that of naive small crayfish in the pairings with naive large crayfish (Table 1B, Fisher’s exact test, *p* = 0.007).

### Effect of Octopamine Injection

Since octopamine is known to have opposing effects to serotonin in many arthropods [22], octopamine at various concentrations was injected into the pericardial sinus of naive crayfish 10 min prior to pairings with smaller naive crayfish (Fig. 3B). As untreated control pairings, large crayfish injected with saline alone won in 75% (9 of 12) of pairs. Only one saline-injected large crayfish was beaten by an untreated small crayfish, while the remaining 2 pairs showed no dominant-subordinate relationship after 45 min of pairing. When 0.1 µM or 0.5 µM octopamine was injected into naive large crayfish, treated animals won 63% (5 of 8) of pairs with 0.1 µM octopamine and 60% (6 of 10) of pairs with 0.5 µM octopamine. There was no statistical difference in winning probability compared to saline-injected large crayfish (Fisher’s exact test, *p* = 0.642 and 0.652, respectively). The winning probability of larger naive animals significantly decreased when 1 µM octopamine was injected. Octopamine-injected large animals won only 20% (2 of 10) of pairs, and were statistically different from saline-injected crayfish (Fisher’s exact test, *p* = 0.030). Despite the larger animals having a physical advantage their winning probability was considerably less than naive large animals shown in Figure 1A (Table 2B, Fisher’s exact test, *p* = 0.001 against naive large crayfish and *p* = 0.538 against subordinate large crayfish in pairings with naive small animals).

0.1 µM tyramine, a precursor of octopamine, was also injected into naive large crayfish 10 min prior to the pairings with naive small crayfish (n = 10). The winning probability of tyramine-injected large crayfish was 30% and was significantly lower than that of naive large crayfish in the pairings with naive small crayfish (Table 2B, Fisher’s exact test, *p* = 0.006).

The total number of fights during agonistic encounters in the first 30 min and the average duration of individual fight of each pairings are shown in Figure 3C and D, respectively. Pairings of saline-injected naive small (Fig. 3A) and naive large (Fig. 3B) crayfish were summed as a saline group, and 1 µM and 2 µM serotonin-injected pairings were summed as a serotonin group, since there were no significant differences between data in each group. The number of fights in the pairings between saline-injected crayfish and untreated naive crayfish was 6.7±1.0 and the average duration of an individual fight was 77.1±12.1 s. Those of pairings between 1–2 µM serotonin-injected small crayfish and untreated naive large crayfish were 4.1±0.5 and 120.2±18.9 s, while those of pairings between 1 µM octopamine-injected large animals and untreated naive small crayfish were 10.2±2.0 and 28.1±5.0 s. The number of fights of serotonin-injected pairings was significantly less than of saline-injected pairings (Mann-Whitney rank sum test, *p* = 0.044) and octopamine-injected pairings (Mann-Whitney rank sum test, *p*<0.001), while the individual fight duration of octopamine-injected pairings was significantly shorter than of other pairings (Mann-Whitney rank sum test, *p* = 0.004 against saline-injected pairings and *p*<0.001 against serotonin-injected pairings). Thus, the injection of serotonin and octopamine affected the outcome of agonistic bouts that were similar to the winner and loser effects of previous experiences of agonistic encounters.

### Effect of Serotonin Blocker

**Figure 4:**
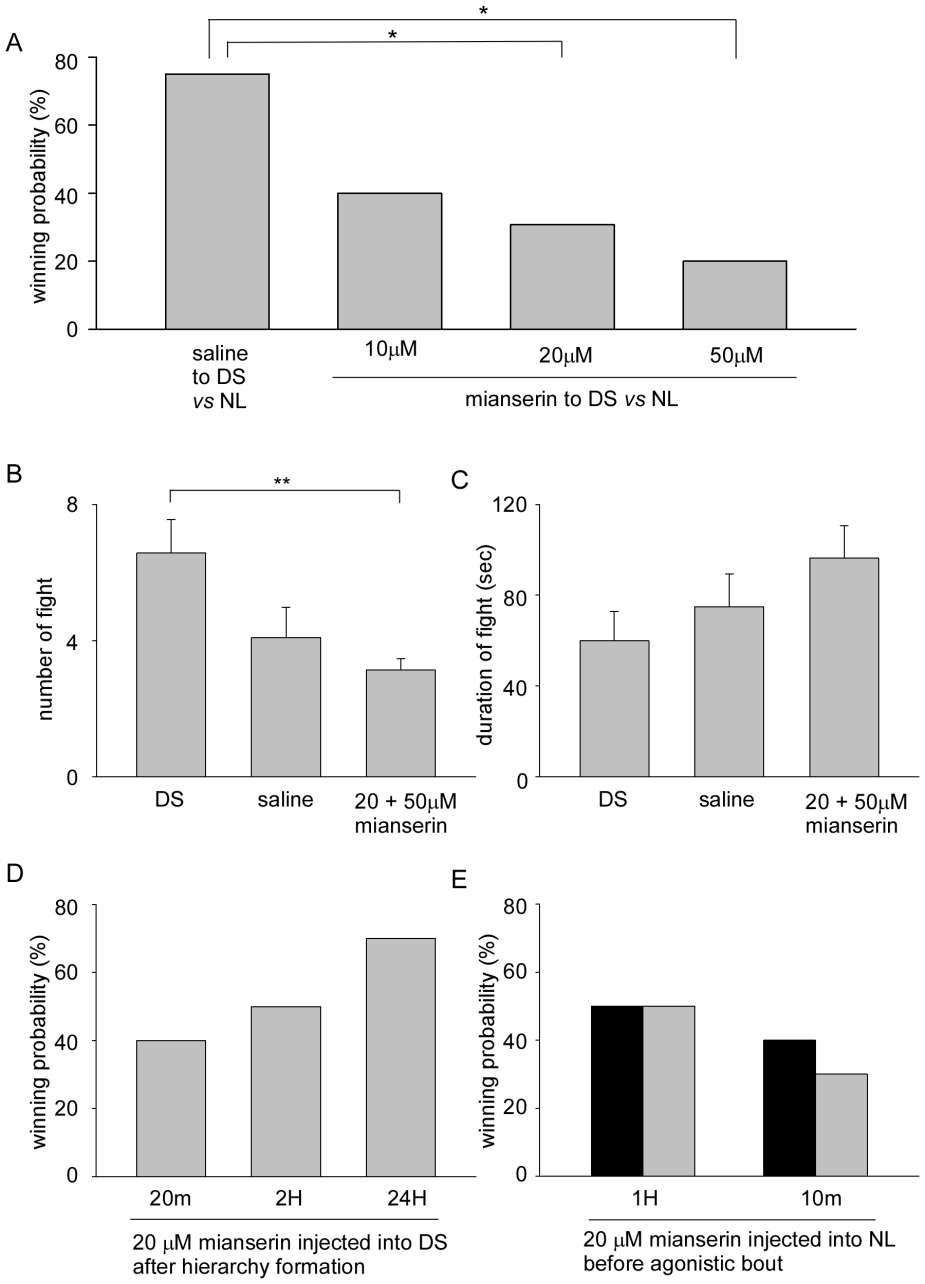
Effect of mianserin on agonistic bouts. A, Winning rates of dominant small crayfish injected mianserin at various concentrations in pairings with naive large crayfish are plotted. Mianserin was injected into dominant animals immediately after establishment of dominant-subordinate formation, and then paired with naive large crayfish after overnight isolation. Asterisks indicate that the winning rate differed significantly (Fisher’s exact test, *p<0.05). B, The number of fights of pairings during the first 30 min of agonistic bouts. C, The average duration of individual fights of pairings during the first 30 min of agonistic bouts. In B and C, data of pairings between dominant small and naive large animals (Left) are from Fig. 2 that sum the data of both winning and losing dominant small crayfish. On the right, 20 and 50 µM mianserin injected into dominant small crayfish are also summed. The asterisk in C indicates that the number of fights differed significantly (Mann-Whitney rank sum test, **p<0.01). D, Effect of timing of mianserin injection into dominant small crayfish after establishment of social rank. The percentages of winning outcomes of agonistic bouts of mianserin-injected small crayfish are plotted. E, Effects of mianserin-injected naive large crayfish 1 hr or 10 min before pairing with naive small crayfish. The percentages of winning outcomes of agonistic bouts of mianserin-injected naive large crayfish are plotted as black bars and those of untreated naive small crayfish are plotted as grey bars.

Mianserin is a known blocker of serotonin or octopamine receptors in invertebrates [37], [42]. Following formation of a dominance order, mianserin was injected into winning crayfish. These dominant crayfish were re-isolated overnight, and then paired with naive crayfish that were larger in size (Fig. 4). When saline alone was injected into dominant crayfish, the treated dominant crayfish won in 75% (9 of 12) of pairings with naive large crayfish the following day. The winning probability of dominant small crayfish decreased after injection of 10 µM mianserin to 40% (4 of 10 pairings). As the concentration of mianserin was increased, more dominant small crayfish were beaten by naive large crayfish. Only 31% of dominant crayfish (4 of 13 pairings) won following the injection of 20 µM mianserin, and 20% of dominant crayfish (2 of 10 pairings) won following the injection of 50 µM mianserin. These were significantly different from saline-injected dominants (Fisher’s exact test, *p* = 0.047 with the injection of 20 µM mianserin and *p* = 0.030 with the injection of 50 µM mianserin). The winning probabilities of the mianserin-injected dominant crayfish were significantly lower than that of control pairings between dominant small and naive large crayfish (Table 1C, Fisher’s exact test, *p* = 0.038 with the injection of 20 µM mianserin and *p* = 0.020 with the injection of 50 µM mianserin). To examine whether mianserin acted as serotonin blocker, 10 µM fluoxetin [26], serotonin reuptake inhibitor, was injected into winning crayfish following formation of a dominance order. These fluoxetin-injected dominant crayfish were re-isolated overnight, and then paired with naive crayfish that were larger in size. 100% of fluoxetin-injected dominant small crayfish won in all 5 pairings that suggested increase in amount of serotonin reinforced winner effect, since winning probability of untreated dominant small animals shown in Figure 1A was 70% (Fisher’s exact test, *p* = 0.289). Thus, the winning effect of previous agonistic encounters was related to serotonin level and mianserin acted as serotonin blocker to disturb the process of winner effect.

The total number of fights during agonistic encounters in the first 30 min and the average duration of an individual fight of each pairing are shown in Figure 4B and C, respectively. Both winning and losing dominant small crayfish in pairings with naive large crayfish (data were from Fig. 2) were summed as a DS group, and mianserin at 20 µM and 50 µM injected pairings were summed as a mianserin group. The number of fights in the pairings between dominant small and naive large animals was 6.6±1.0 and the average duration of an individual fight was 59.8±13.0 s. In the pairings between saline-injected dominant small and untreated naive large animals, the mean number of fights was 4.1±0.9 and the fight duration was 74.8±14.5 s. Those of pairings between 20–50 µM mianserin-injected dominant small crayfish and untreated naive large crayfish were 3.1±0.3 and 96.3±14.4 s. Both were different from those of control pairings (Mann-Whitney rank sum test; *p* = 0.009 for number of fights and *p* = 0.051 for fight duration). In the losers of mianserin-injected dominant small crayfish, the number of fights was 3.5±0.4 that was significantly smaller compared to that of pairings between the losers of the dominant small animals and naive large animals (11.2±2.2 as Fig. 2B) (Mann-Whitney rank sum test, *p*<0.001). Thus, the aggressive motivation of dominant small crayfish was decreased by the injection of mianserin.

Figure 4D shows the effect of 20 µM mianserin that was injected at various times after the establishment of dominance order upon outcomes of agonistic bouts in pairings with naive large crayfish the following day. When mianserin was injected 20 min after the establishment of dominance order, dominant small crayfish won in 40% (4 of 10) of pairings. The winning probability was 50% for mianserin injected 2 hr after the determination of social order. Although the winning probabilities of mianserin treated crayfish were lower than that of untreated dominant small animals ( = 69.6%, Fig. 2A), there was no statistically significant difference (Fisher’s exact test, *p* = 0.139 in mianserin injected 20 min after the determination of social order and *p = *0.433 in mianserin injected animals after 2 hr). The winning probability of dominant small crayfish was 70% following mianserin injection 24 hr after the establishment of dominance order that was similar to untreated winners (Fisher’s exact test, *p* = 1.0). Next, we injected 20 µM mianserin into naive large animals 1 hr or 10 min prior to pairings with naive small crayfish (Fig. 4E). 50% of larger animals won in 10 pairings following injection of mianserin 1 hr prior to pairings, while 40% (4 of 10) of larger crayfish won following mianserin injection 10 min prior to pairings (3 larger animals were beaten and the remaining 3 animals drew). The winning probability of mianserin-injected large crayfish considerably decreased from 83% in untreated naive large crayfish to less than 50% (Table 2C, Fisher’s exact test, *p* = 0.035 in mianserin injected 10 min prior to pairings and *p = *0.090 in mianserin injected 1 hr prior to pairings). Thus, serotonin also affected aggressiveness during agonistic bouts.

### Effect of Octopamine Blocker

**Figure 5:**
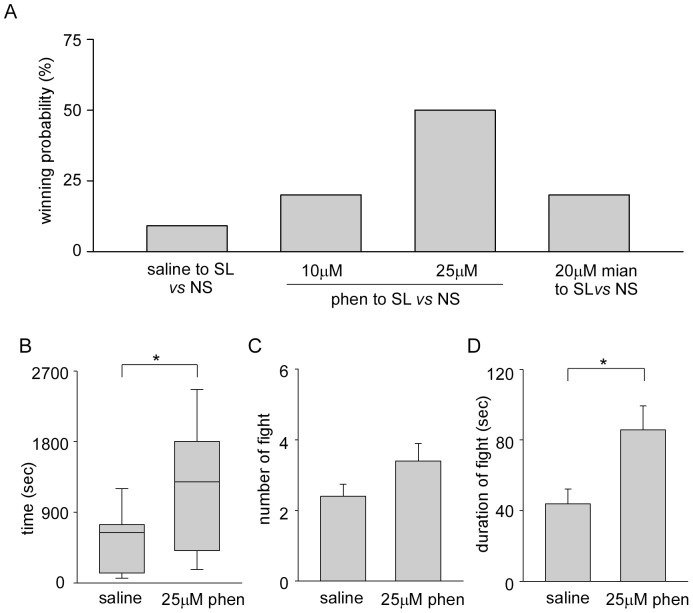
Effect of phentolamine on agonistic bouts. A, Winning rates of subordinate large crayfish injected with saline, phentolamine of 10 µM or 25 µM and 20 µM mianserin in pairing with naive small crayfish are plotted. Physiological saline, phentolamine or mianserin was injected into subordinate animals immediately after establishment of dominant-subordinate formation, and then paired with naive small crayfish after overnight isolation. B, The time during which the dominant-subordinate relationship was determined in both pairings between saline-injected subordinate large crayfish and untreated naive small crayfish and pairings between 25 µM phentolamine-injected subordinate large crayfish and untreated naive small crayfish. Box plots show median (solid black line), interquartile range (box length), and minimum and maximum values (error bars). Asterisk indicates that the time for social rank formation was different statistically (Log-rank test, *p<0.05). C, The number of fights of pairings during the first 30 min of agonistic bouts. D, The average duration of individual fights of pairings during the first 30 min of agonistic bouts. Asterisk indicates that the duration of responses differed significantly (Mann-Whitney rank sum test, *p<0.05).

Phentolamine is a known octopamine receptor blocker in invertebrates [38]. Just after the determination of dominance order, phentolamine or mianserin was injected into losing crayfish. Those subordinate crayfish were re-isolated overnight, and then paired with naive crayfish of smaller size (Fig. 5). When saline was injected alone into subordinate crayfish just after formation of dominance order, large treated crayfish won in 9% (1 of 11) of pairings with naive small crayfish the following day. The winning probability of subordinate large crayfish slightly increased after the injection of 10 µM phentolamine to 20% (2 of 10 pairings), but was not statistically different from saline-injected animals (Fisher’s exact test, *p* = 0.571). When 25 µM phentolamine was injected, the winning probability increased to 50% (5 of 10 pairings) and was almost statistically different from saline-injected animals (Fisher’s exact test, *p* = 0.056). The winning probability of 20 µM mianserin-injected subordinate large crayfish was 20% (2 of 10 pairings) and was not statistically different from saline-injected animals (Fisher’s exact test, *p* = 0.571). Thus, the loser effect of previous agonistic encounters was cancelled by the injection of 25 µM phentolamine (Table 2D, Fisher’s exact test, *p* = 0.018). Phentolamine at a 25 µM concentration was also injected into dominant small animals just after the determination of dominance order. They won in 80% (4 of 5) of pairings with naive large animals on the following day. Thus, phentolamine did not affect the winner effect of the dominant small crayfish (Table 1C, Fisher’s exact test, *p* = 1.0).

The average length of the decision time for dominance formation in the pairings between saline-injected subordinate large and naive small animals was 511.5±121.9 s (Fig. 5B left), which was similar to that of pairings between untreated subordinate large and naive small animals. On the other hand, the decision time in the pairings between 25 µM phentolamine-injected subordinate large animals and naive small animals was 1165.1±252.7 s (Fig. 5B right), and was significantly longer than that of saline-injected pairings (log-rank test, *p* = 0.016). The number of fights in the pairings between saline-injected subordinate large and naive small animals was 2.4±0.3 and that of pairings between 25 µM phentolamine-injected subordinate large and naive small crayfish was 3.4±0.5 (Fig. 5C). The average duration of individual fights in the former pairings was 43.8±8.5 s while that of the latter pairings was 85.8±13.5 s (Fig. 5D). When 25 µM phentolamine was injected, the fight duration became significantly longer than that of the saline-injected pairings (Mann-Whitney rank sum test, *p* = 0.017) and untreated pairings shown in Figure 2 (Mann-Whitney rank sum test, *p* = 0.002). When 25 µM phentolamine was injected into naive small crayfish 10 min prior to the pairings with naive large animals, phentolamine-injected small crayfish won in 30% (3 of 10) of pairings. This was not statistically different to the winning probability of untreated naive small animals in pairings with naive large animals (Table 1B, Fisher’s exact test, *p* = 0.336).

We also examined the effect of epinastine, another octopamine blocker [39], upon loser effect. Just after the determination of dominance order, 25 µM epinastine was injected into losing crayfish. These epinastine-injected subordinate crayfish were re-isolated overnight, and then paired with naive crayfish of smaller size. The winning probability of epinastine-injected animals also increased to 50% (3 of 6 pairings) that was similar to the winning probability of 25 µM phentolamine-injected animals (fisher’s exact test, *p* = 1.0).

## Discussion

### Social Experience Affects Aggressive Motivation of Crayfish

The relative size of animals is directly related to dominance hierarchy formation in crustaceans, including crayfish [43], [44], [45], [46], [47], [48], [49]. As the results of our previous study showed, more than 80% of winning crayfish had longer bodies and/or chelae length, and winners were usually heavier in mass, even if their differences were less than 5% of the losing opponents [50]. Previous winners, however, won significantly more encounters than previous losers during their subsequent conflicts. These social experiences are known as winner and loser effects and affect the outcomes of agonistic bouts across different animal taxa (for review see [51] ). In lobsters and crayfish, winner effects are known to last for 1–2 weeks [36], [52]. We have demonstrated clearly in this study that the winners of the first pairings were more likely to win their second fight with inexperienced naive crayfish, even if opponents were 3–7% larger in size. Similarly, losers of the previous pairings were frequently beaten by smaller naive crayfish in the second fight, even if losers had a physical advantage. In the second fights between dominant animals, larger crayfish won significantly more often. The decision time to the establishment of a new dominant-subordinate relationship and fight intervals became shorter suggesting aggressiveness would be escalated in winning animals. By contrast, larger crayfish seemed to have no advantage in second fights between subordinate animals. Larger animals won in only 30% of trials and no clear social order was determined in the remaining 50%. In the latter pairings, both crayfish avoided encounters each other and few fights with a very short duration were observed. These results suggest that aggressive motivation could be reduced in losing crayfish.

### Aminergic Control of Social Status

Serotonin in the brain has been studied extensively for its role in the neurobiological basis of aggression in vertebrates. Increases in the level of serotonin have frequently been reported to reduce aggressive behavior in fish [53], lizards [54] and rodents [17], [21], [55], [56], [57], although some studies suggest that serotonin promotes aggression in vervet monkeys [58], dogs [59] and mice [60]. For example, aggressive behavior is decreased by treatment with 5-TH 1A and 5-HT1B receptor agonists in rats [57] and by treatment with serotonin reuptake inhibitor in lizards [54]. Furthermore, aggression is increased in 5-HT1B receptor knock-out mice [55]. By contrast, serotonin in crustaceans is known to enhance aggressive motivation [61], [62]. Injection of serotonin elicits a dominant-like posture in both lobsters and crayfish [22], [23], [35] and aggressive displays in squat lobsters [24]. Injection of serotonin into small subordinate crayfish decreases the likelihood of retreat from dominant large opponents with large size asymmetries (>30%) and increases the duration of fighting [25], [26]. Recently, serotonin is also reported to increase the hemolymph glucose level and the crustacean hyperglycemic hormone (CHH) that modulate aggression of crayfish [63], [64]. Octopamine is known to elicit opposing, subordinate-like posture in crayfish [22] and behaviors typical of subordinate animals in squat lobsters [24]. In crabs, the injection of octopamine decreases fight duration [28]. This study clearly indicates that serotonin enhanced aggressive motivation while octopamine reduced it, since serotonin-injected small crayfish was more likely to win in pairings with untreated large crayfish that had a physical advantage. Furthermore, octopamine-injected large crayfish was less likely to win in pairings with small crayfish that had a physical disadvantage. Furthermore, injection of octopamine blockers cancelled loser effects. This is first report to clearly demonstrate a role for octopamine to reduce aggression, although octopamine is thought to enhance aggression in crickets [65].

### Serotonin and Octopamine Mediate Winner and Loser Effects

The winner and loser effects are widespread across different animal taxa. Despite their ubiquity, the mechanisms underlying winner and loser effects are still poorly understood. In cichlids, androgen level is a causal mediator of a winner effect [4]. Treatment with an anti-androgen blocks the winner effect, whereas androgen administration fails to reverse the loser effect, suggesting an involvement of androgens on the winner but not on the loser effect. In this study, exogenous serotonin and octopamine mimicked the winner and loser effect, respectively. Injection of serotonin into naive crayfish tended to lead to winning in the pairings with larger un-experienced opponents, which was similar to the pairings between dominant small and naive large animals. By contrast, injection of octopamine into naive crayfish was likely to lead to losing in the pairings with smaller opponents, that was similar to the pairings between subordinate large and naive small animals. Injection of the serotonin precursor, 5 HTP into naive small crayfish also increased the winning probability in the pairings with larger naive opponents, while injection of the octopamine precursor, tyramine into naive large crayfish also decreased the winning probability in the pairings with smaller naive opponents. These results indicate that endogenous serotonin and octopamine could be responsible for the outcomes of agonistic encounters. Furthermore, the winner effects of dominant crayfish were cancelled by the injection of mianserin, an antagonist of serotonin receptors [37] and were reinforced by the injection of fluoxetin, serotonin reuptake inhibitor [26], just after the establishment of social order of the first pairings. Injection of an octopamine channel blockers, phentolamine [38] and epinastine [39], by contrast, cancelled the loser effects. These results strongly indicate that serotonin and octopamine could mediate the winner and loser effects respectively. According to which winning and losing experience, status-dependent changes would occur in internal state. An increase in serotonin levels in dominant winners and/or increases in octopamine levels in subordinate losers could affect the outcomes of subsequent contests. Thus, social experiences could affect aggressive motivation mediated through endogenous serotonin and octopamine levels. In crayfish and lobsters, serotonergic and octopaminergic neurons are widely distributed in the central nervous system [66], [67], [68]. Sneddon et al. (2000) shows that the serotonin concentration in a crab hemolymph increased while octopamine concentration decreased in winners from resting values, but in losers octopamine levels increased from resting concentrations [69]. Yeh et al. (1996) also shows that serotonin reversibly enhanced the response of sensory stimulation of the crayfish lateral giant neuron (LG) in socially dominant crayfish and reversibly inhibited it in subordinate animals [70]. Furthermore, Cattaert et al. (2010) showed that the excitability of the leg postural circuit of the crayfish and its response to serotonin was enhanced in dominants but decreased in subordinates [71]. These results correlate with our findings.

Mianserin is used as a serotonin antagonist in both vertebrates [72], [73] and invertebrates, including the pond snail [37] and insects (locust [74]; silkmoth [75]; cockroach [76]), although some studies have used mianserin as a channel blocker of octopamine, such as in the crab [39] and honey bee [42]. The present data support the notion that mianserin acts as a serotonin blocker in this system since the injection of mianserin into subordinate crayfish had no significant effect upon the outcomes of following conflicts though octopamine antagonists, phentolamine and epinastine cancelled the loser effect of subordinate animals. Furthermore, injection of fluoxetin, serotonin reuptake inhibitor reinforced the winner effect while injection of mianserin cancelled it. Two of potentially five serotonin receptors in crayfish, 5-HT1α and 5-HT2β, have been cloned and pharmacologically characterized [77]. The protein domains involved in G protein coupling are conserved with mammalian 5-HT type 1 (5-HT1) and type 2 (5-HT2) receptors. A vertebrate 5-HT1 agonist inhibits the response of LG to sensory stimulation in both dominant and subordinate crayfish, while a vertebrate 5-HT2 agonist increases the sensory response of LG in both dominant and subordinate animals [32], [70]. Since mianserin is known to be a 5-HT2 antagonist in vertebrates [73], asymmetrical expression of 5-HT2β receptors between becoming dominants and subordinates could be related to social status of the crayfish. Further pharmacological analyses using specific serotonin agonists and antagonists are necessary to clarify this point. As delayed injection of mianserin after the establishment of dominance order, mianserin became less effective. This suggests that serotonin was not necessary to maintain the winning status of dominant crayfish. The majority of serotonin and octopamine receptors belong to a superfamily of G-protein coupled receptors and their effects are mediated by second messengers [77], [78], [79], [80]. Thus, serotonin and octopamine could activate downstream second messenger system, e.g. cAMP [81] and IP_3_ [82] cascades to maintain dominant and subordinate status.
